# Evaluation of Midwife‐Led Colposcopy for Female Genital Schistosomiasis Screening at Primary Level of Care in Rural Madagascar: A Cross‐Sectional Study

**DOI:** 10.1111/tmi.70049

**Published:** 2025-10-30

**Authors:** Pia Rausche, Jean‐Marc Kutz, Zoly Rakotomalala, Bodo Sahondra Randrianasolo, Paule Donven, Rivo Solotiana Rakotomalala, Alexina Olivasoa Tsiky Zafinimampera, Olivette Totofotsy, Sonya Ratefiarisoa, Ravo Razafindrakoto, Nantenaina Matthieu Razafindralava, Zaraniaina Tahiry Rasolojaona, Jana Hey, Aaron Remkes, Tahinamandranto Rasamoelina, Eva Lorenz, Rapahel Rakotozandrindrainy, Jürgen May, Monika Hampl, Irina Kislaya, Valentina Marchese, Rivo Andry Rakotoarivelo, Daniela Fusco

**Affiliations:** ^1^ RG Implementation Research Bernhard Nocht Institute for Tropical Medicine Hamburg Germany; ^2^ German Center for Infection Research (DZIF) Hamburg ‐ Lübeck ‐ Borstel ‐ Riems Germany; ^3^ Centre Hospitalier Universitaire Androva Mahajanga Madagascar; ^4^ Association K'OLO VANONA Antananarivo Madagascar; ^5^ University Fianarantsoa Fianarantsoa Madagascar; ^6^ Centre D'infectiologie Charles Mérieux Antananarivo Madagascar; ^7^ Department of Infectious Diseases Epidemiology Bernhard Nocht Institute for Tropical Medicine Hamburg Germany; ^8^ University Antananarivo Antananarivo Madagascar; ^9^ Tropical Medicine I University Medical Center Hamburg‐Eppendorf (UKE) Hamburg Germany; ^10^ Department of Obstetrics and Gynaecology St. Elisabeth Hospital Cologne‐Hohenlind Germany; ^11^ University Hospital of Düsseldorf Düsseldorf Germany

**Keywords:** colposcopy, diagnostic accuracy, female genital schistosomiasis, midwifery, neglected diseases, primary health care

## Abstract

**Introduction:**

Female genital schistosomiasis is a condition with a complex diagnosis and severe consequences such as infertility. In the absence of a reliable biomarker, in endemic settings the World Health Organization recommends colposcopy as a diagnostic tool for the detection of female genital schistosomiasis lesions. Nevertheless, it is seldom performed in low‐resource contexts due to a lack of expertise or insufficient infrastructure. This study aims to assess Female Genital Schistosomiasis colposcopy at the primary level of care, evaluating its diagnostic accuracy in reference to gynaecologist diagnosis in a highly endemic context.

**Materials and Methods:**

This is a secondary analysis of a cross‐sectional study conducted in the Boeny region of Madagascar, which collected colposcopy images and Female Genital Schistosomiasis decision at the primary health care level with re‐evaluation by gynaecologists. Statistical analysis using R included descriptive statistics, measures of diagnostic accuracy with 95% confidence intervals and binary Poisson regression with robust standard errors, while reporting followed the STARD statement.

**Results:**

Among 495 included participants, a high sensitivity [96.4% (95% CI 93.7–98.0)] and relatively low specificity [28.7% (95% CI 21.8–36.5)], with a fair agreement [*κ* 0.30 (95% CI 0.22–0.39)], was observed for midwife‐led colposcopy. Practice of midwives (3.5 months) was associated with reduced concordance [APR 0.88 (95% CI 0.79–0.98)] and specificity [APR 0.27 (95% CI 0.15–0.49)]. The environment of one health care centre negatively influenced concordance and specificity of midwife‐led colposcopy.

**Conclusion:**

Midwives can detect female genital schistosomiasis with high sensitivity but limited specificity when compared to expert gynaecologists, revealing variation in performance between environments as well as the influence of practice and workload. This study suggests that implementing midwife‐led colposcopy at primary care level for female genital schistosomiasis screening is feasible but requires appropriate quality assurance measures.

**Abbreviations**
APR: adjusted prevalence ratio
CPR: Crude prevalence ratio
FGS: female genital schistosomiasis
HPV: human papilloma virus
NPV: negative predictive value
PHCC: primary health care centre
PID: pseudonymised patient identifier
PPV: positive predictive value
STI: sexual transmitted infection
WHO: World Health Organization

## 1 Introduction

Female Genital Schistosomiasis (FGS) is the gynaecological consequence of a persistent infection with *Schistosoma haematobium*, a vector‐borne trematode causing the urogenital form of human schistosomiasis [1, 2]. FGS is caused by the entrapment of *Schistosoma* eggs in genital tract tissue. The pathogenesis of FGS is driven by the host inflammatory response, inducing the formation of granulomatous reactions surrounding the deposited ova [3]. Left untreated, FGS can produce mucosal alterations such as fibrosis and ulceration, and may lead to complex gynaecological syndromes, including pelvic pain, infertility, and increased susceptibility to the human immunodeficiency virus [4, 5]. Symptoms of FGS often resemble those of sexually transmitted infections (STIs), including vaginal discharge, pain, or irregular bleeding. This, together with limited knowledge of the condition [6], leads to misdiagnosis and underreporting of FGS, resulting in inaccuracies in the global epidemiology. Current estimates suggest that between 40 and 56 million women and girls are affected by FGS worldwide [4, 7], with studies reporting positivity rates of up to 60% in areas with a high burden of *S. haematobium* infection [8].

The diagnosis of FGS is complex. The gold standard diagnostic is the histopathology of tissues collected from the genital tract. This diagnostic method requires highly specialised personnel and it carries certain procedural risks, rendering it unsuitable for use in resource‐limited, endemic settings [9]. Currently, there are no standardised molecular procedures for the detection of the condition [10]. The mere presence of eggs in the urine is not necessarily indicative of FGS, nor is the presence of FGS lesions a clear indicator of active infection with *S. haematobium* eggs in the urine, making urine microscopy unsuitable for the diagnosis of FGS [11]. In absence of a reliable biomarker in endemic settings, the World Health Organization (WHO) recommends colposcopy as a diagnostic tool for the detection of FGS lesions [5]. Colposcopy is the magnified visual examination of the cervicovaginal mucosa, requiring trained personnel and specialised equipment to be performed. Inter‐ and intra‐operator reliability as well as limited specificity are frequently reported for the procedure [12, 13]. According to the WHO definition, a woman can be diagnosed with FGS in the presence of (a) abnormal blood vessels or (b) rubbery papules or (c) homogeneous yellow sandy patches or (d) grainy sandy patches or simultaneous occurrence of these lesions in the genital tract [5].

Despite its limitations, colposcopy is widely used in clinical practice especially for the screening of cervical cancer in combination with visual inspection utilising acetic acid and Lugol's iodine solution [14]. Studies have shown its applicability and good impact on women's health at primary level of care [15], especially for the screening and management of cervical cancer [16]. In this view, integrated approaches targeting women's health more broadly have been suggested to improve the management of FGS and overall women's health in FGS endemic countries [17].

Madagascar is among the countries with the highest prevalence of FGS worldwide. Furthermore, the country's circumstances create significant barriers regarding accessibility to healthcare services. Especially for women and populations living in rural areas, services like colposcopy are almost never available at any level of care [18, 19]. The establishment of specialised women's health services at the primary care level could contribute to the adoption of preventive practices [20].

Therefore, this study aims at assessing the suitability of colposcopy for the screening of FGS at the primary level of care through the evaluation of its diagnostic accuracy and inter‐rater reliability in a highly endemic context, such as rural Madagascar. The main objective of the study is to investigate the performance of midwife‐led colposcopy‐based FGS diagnosis at the primary level of care in comparison to an expert gynaecologist diagnosis.

## 2 Methods

### 2.1 Study Design and Area

This cross‐sectional study is a secondary analysis of a study conducted in the Boeny region of Madagascar with a prevalence of FGS above 60% and an estimated population of 543,200 inhabitants [8]. Study sites were three primary health care centres (PHCCs), including two rural PHCCs: Antanambao‐Andranolava (15°58′00″S, 46°41′00″E, 3000 inhabitants), Ankazomborona (16°06′50″ S, 46°45′24″ E, 23,000 inhabitants, including periphery), and one peri‐urban PHCC, Marovoay (16°06′40″ S, 46°38′38″ E, 34,000 inhabitants).

### 2.2 Recruitment and Eligibility Criteria

Recruitment took place between April and December 2022. Through a convenience sampling, participants were recruited in the context of a gynaecological investigation performed by trained nurses and midwives at the PHCCs. Eligibility for inclusion in the analysis was (a) adult women of reproductive age (18–49); (b) capacity to provide informed consent; (c) fluent in French or Malagasy; (d) absence of pregnancy; and (e) no history of praziquantel treatment within the preceding year, in order to minimise possible confounding effects of recent therapy on lesion appearance.

### 2.3 Gynaecological Investigation and Image Collection

Inspection of the cervix for signs of FGS was performed using binocular Centrel‐S12 colposcopes (Centrel Srl, Italy) and conducted by seven trained midwives and nurses routinely working at the PHCCs as described in Kutz et al. [8]. Initial training consisted of a one‐week training and concluding 2 weeks support of an experienced gynaecologist. Before the start of image collection, midwives who worked in the previous colposcopy campaign (*n* = 5) took part in follow‐up training, while new midwives in the program (*n* = 2) received introductory training and were mentored in a peer‐to‐peer approach during the data collection by a midwife who participated in the previous trainings. Five of the midwives participated for the whole duration of the study; one midwife was replaced during the data collection in the PHCC of Antanambao‐Andranolava. Images of the cervix were taken using the USB DL1 digital camera (Centrel, Italy) and stored with a pseudonymised patient identifier (PID) on password‐protected hard drives. In case of the presence of any possible FGS signs, women were offered treatment with 40 mg/kg praziquantel. If no colposcopy was performed or no image was collected, women were excluded from this analysis.

### 2.4 Data Collection and Data Management

Data were collected through a structured paper case‐report form, in which demographic information and information on gynaecological symptoms were provided, followed by image collection during colposcopy. All case‐report forms were assigned a pseudonymised PID to ensure secure attribution of participant and image. Trained data clerks in Mahajanga, Madagascar, performed double data entry, with regular data checks conducted by the data manager. All data were entered into a REDCap database hosted on a secure server at the Bernhard Nocht Institute for Tropical Medicine in Hamburg, Germany [21, 22].

### 2.5 Diagnosis

Midwives determined FGS positivity based on the presence of characteristic FGS signs as defined in the WHO FGS Pocket Atlas for Clinical Healthcare Professionals [5]. Reference diagnosis was established in a two‐stage blinded assessment of the cervical images through two independent gynaecologists, experienced in the field of FGS. Images were evaluated between August and September 2023, using a predesigned Microsoft Excel matrix. Previous index diagnosis was not known to the gynaecologists. Categories evaluated by the gynaecologists included: (a) the presence of four characteristic FGS signs; (b) image quality, rated on a scale from 1 (lowest quality) to 10 (highest quality); (c) descriptions of image limitations; and (d) additional gynaecological findings, such as cervical lesions unrelated to FGS. FGS positivity was defined as the presence of at least one FGS sign reported by both gynaecologists. Images were classified as positive, negative or discordant in cases of disagreement. Discordant images from the first evaluation round were re‐assessed by the gynaecologists during a moderated consensus meeting. Images lacking consensus after the second evaluation round were excluded from the study.

### 2.6 Variable Description and Statistical Analysis

Data analysis was structured into two macro domains: (A) participant characteristics and (B) procedure characteristics. Macro domain A comprised the micro domains age group, presence and type of gynaecological symptoms. Macro domain B contains the micro domains of environmental and methodological factors, as well as midwives' training and practice. The location of the PHCC was considered an environmental factor. As a proxy for the methodological factors, the image quality (averaged between the two gynaecologists) was considered, based on the assumption that lower image quality reflects a more rushed procedure of the midwives, with less time to adjust for such factors. Quality at or below the median was categorised as lower quality and above the median as higher quality.

Practice duration (in months) was standardised to midwife's individual start date in the current colposcopy campaign. Descriptive statistics were reported for all micro domains, with categorical variables summarised as absolute and relative frequencies, rounded to one decimal point.

To assess the performance of the FGS signs detection by midwives at PHCC, this study analysed: (a) diagnostic concordance; (b) sensitivity; (c) specificity; (d) positive predictive value (PPV); (e) negative predictive value (NPV) using the *diagnostic* function from the *ThresholdROC* R package [23]. The inter‐rater agreement between midwives and reference was presented as Cohen's kappa (*κ*) with interpretation after Landis and Koch, estimated via the *Kappa* function of the *vcd* package [24, 25]. Estimates were presented with 95% confidence intervals (95% CI) for the overall sample and stratified by each of the domains previously described.

To identify factors associated with concordance, specificity and sensitivity, univariate and, where appropriate, multivariate binary Poisson regression with robust sandwich standard errors was performed; crude prevalence ratios (CPR) and adjusted prevalence ratios (APR) were estimated. Inclusion in the regression model followed a data‐driven approach, based on pre‐testing with chi‐square and Fisher's exact test, combined with expert‐based selection. Variables were included if they were significant in pre‐tests or, in the case of the screening practice, supported by a strong hypothesis of influence on the outcome. All analysis was performed using R version 4.3.2 (R Foundation for Statistical Computing, Vienna, Austria).

### 2.7 Sample Size

The sample size estimation was based on an expected prevalence of 50%, a marginal error of ±5%, and anticipated performance of 90% sensitivity and 30% specificity, yielding an estimated requirement of 645 participants.

### 2.8 Ethical Considerations

This study was approved by the Ethic Committee Hamburg State Medical Chamber (protocol number PV7309‐4707_2‐BO‐ff) and the National Ethic Committee of Madagascar (protocol number 052/MSANP/SG/AGMED/CERBM). Participants had the right to withdraw informed consent and study participation at any time without giving reasons. Financial compensation for participants was restricted to the reimbursement of transportation costs. Procedures for following up suspected pathologies different from FGS were implemented as reported in Kutz et al. [8].

## 3 Results

### 3.1 Participants, Images and Staff Characteristics

**FIGURE 1:**
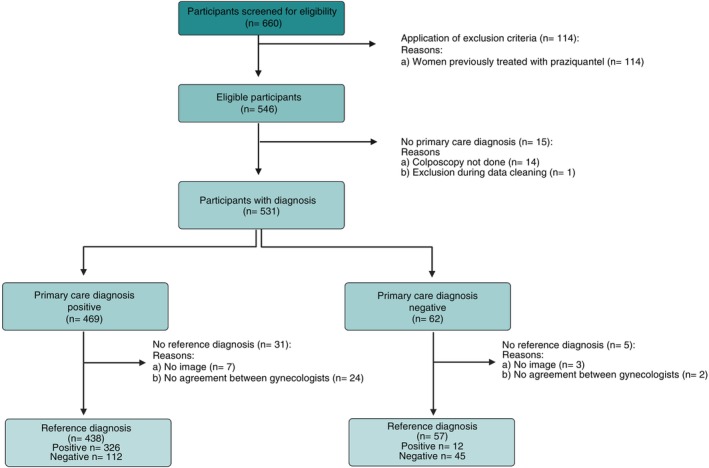
STARD inclusion flowchart displaying the in‐ and exclusion of study participants. Among the 660 women screened for eligibility 114 were excluded due to previous treatment with praziquantel. Among the 546 eligible participants, 15 were excluded due to non‐conducted colposcopy and data cleaning, with 531 participants evaluated at primary level of care. No reference diagnosis was possible in 36 cases, leaving 495 participants for analysis. Created with Biorender.com.

For this secondary analysis, among 660 recruited women, a total of 495 complete diagnostics were analysed (Figure 1) whose characteristics are displayed in Table 1.

**TABLE 1:** Sample characteristics of included images with participant characteristics including age group and gynaecological symptoms, environmental factors, methodological factors, training and practice.

	Overall (*n* = 495)
	*n* (%)
Participant characteristics	
Age group	
18–27	247 (49.9)
28–37	134 (27.1)
38+	114 (23.1)
Gynaecological symptoms	
Yes	333 (67.3)
No	162 (32.7)
Irregular bleeding	126 (25.5)
Vaginal discharge	219 (44.2)
Pain during/after sex	99 (20.0)
Genital itch	178 (36.0)
Procedure characteristics	
Environmental	
Antanambao‐Andranolava	174 (35.2)
Ankazomborona	164 (33.1)
Marovoay	157 (31.7)
Methodological	
Lower image quality	282 (57.0)
Higher image quality	213 (43.0)
Training	
Previous experience	393 (79.4)
Newly trained	102 (20.6)
Practice	
0–2 months	148 (29.9)
3–5 months	224 (45.3)
6–8 months	123 (24.8)

Diagnosis with the reference standard was positive in 68.2% of cases. In total, 49.9% of women were between 18 and 27 years (*n* = 247), while the age groups 28–37 and 38+ were represented by 27.1% (*n* = 134) and 23.1% (*n* = 114), respectively. Among the participants, 67.3% (*n* = 333) self‐reported at least one gynaecological symptom in the past 6 months. The most common symptom was vaginal discharge in 44.2% (*n* = 219), followed by genital itching with 36.0% (*n =* 178) and irregular bleeding for 25.5% (*n* = 126) of the women. The least common symptom was pain after or during sexual intercourse at 20.0% (*n* = 99). The evaluation of the image quality showed 57.0% (*n* = 282) of images classified as lower quality and 43.0% (*n =* 213) images classified as higher quality. Images were collected in 35.2% (*n* = 174) of cases in Antanambao‐Andranolava, 33.1% (*n* = 164) in Ankazomborona, and 31.7% (*n* = 157) in Marovoay. Midwives attending more than one colposcopy training took 79.4% (*n* = 393) of the images. Most images [45.3% (*n* = 224)] were taken when the individual midwives had a practice of 3–5 months, while 29.9% (*n* = 148) were taken at relatively low practice with 0–2 months of active screening and 24.8% (*n* = 123) with a high practice at 6–8 months of screening.

### 3.2 Diagnostic Concordance and Inter‐Rater Agreement

Midwives classified the images with a concordance of 75.0% [*n* = 371 (95% CI 70.9–78.7)] when compared to the reference diagnosis, with an overall fair inter‐rater reliability [*κ* = 0.30 (95% CI 0.22–0.39)]. Among the 338 images considered positive for FGS by gynaecologists, midwives observed FGS signs in 326, leading to a sensitivity of 96.4% (95% CI 93.7–98.0), while only 45 out of 157 images were correctly classified by midwives as negative, with an overall lower specificity of 28.7% (95% CI 21.8–36.5). PPV and NPV were similar, with 74.4% (95% CI 70.0–78.4) and 78.9% (95% CI 65.8–88.2), respectively (Table 2).

**TABLE 2:** Measures of diagnostic accuracy and inter‐rater agreement for the diagnosis of the midwives in reference to the diagnosis of the gynaecologists.

	PHCC+, Ref+	PHCC+, Ref−	PHCC−, Ref+	PHCC−, Ref−
*All images* (*overall*)	326	112	12	45
	*Estimates* (*95% CI*)			
Concordance	75.0% (70.9–78.7)			
Sensitivity	96.4% (93.7–98.0)			
Specificity	28.7% (21.8–36.5)			
Positive predictive value (PPV)	74.4% (70.0–78.4)			
Negative predictive value (NPV)	78.9% (65.8–88.2)			
Cohen's Kappa (*κ*)	0.30 (0.22–0.39)			

### 3.3 Influences on PHCC Diagnostics

**FIGURE 2:**
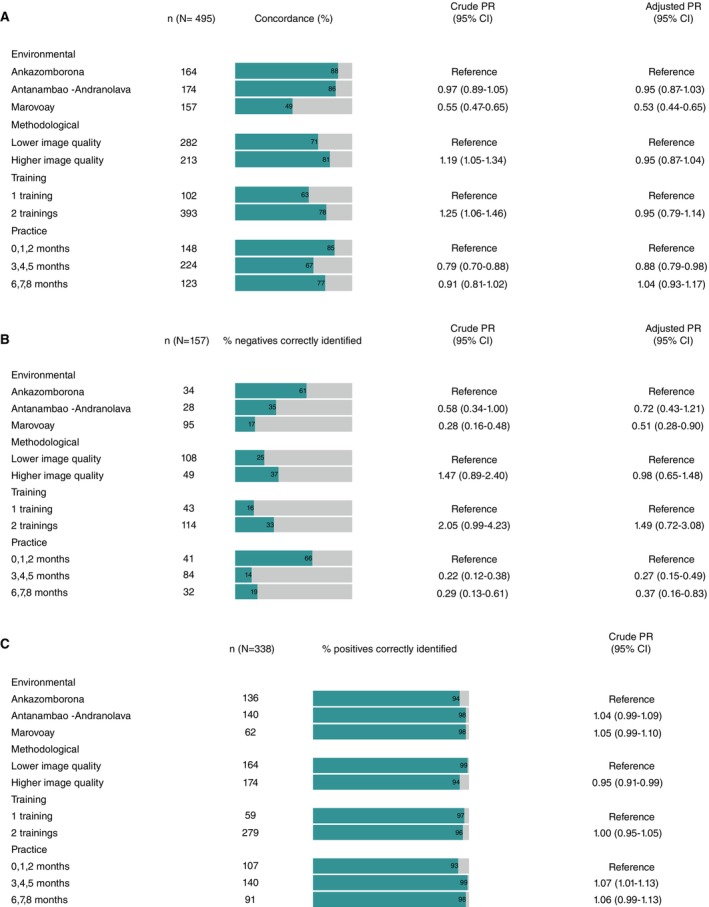
(A) Binary Poisson regression with robust sandwich errors of the overall accurate detection on presence of FGS signs at PHCC (*n* = 495) in comparison to the reference diagnosis, variance inflation factors: Environment 1.6, methodological 1.1, training 1.4, practice 1.1. Crude prevalence ratio (Crude PR) with 95% confidence intervals, adjusted prevalence ratios (Adjusted PR) with 95% CIs. For display, concordance is rounded up to full numbers. (B) Binary Poisson regression with robust sandwich errors of cases where no FGS signs were detected (*n* = 157). Crude prevalence ratio (Crude PR) with 95% confidence intervals, adjusted prevalence ratios (Adjusted PR) with 95% CIs., variance inflation factors: Environmental 1.5, methodological 1.1, training, 1.16, practice 1.18. For display, percentage of correct diagnosis on PHCC level is rounded up to full numbers. (C) Univariate binary Poisson regression with robust sandwich errors of the cases where FGS signs were detected in the reference diagnosis (*n* = 338). For display, percentage of correct diagnosis on PHCC level is rounded up to full numbers.

Due to a very small number of negative images that were determined positive by the gynaecologists (*n* = 12) (Figure 2C), multivariate regression analysis was restricted to concordance (Figure 2A) and specificity (Figure 2B). Concordance varied mostly in the macrodomain of procedure characteristics (Supporting Information S1).

The environments of the PHCC of Ankazomborona reached a concordance (Figure 2A) of 88.4% (95% CI 82.5–92.9), PHCC Antanambao‐Andranolava of 85.6% (95% CI 79.5–90.5) while the PHCC Marovoay presented a concordance of 49.1% (95% CI 41.0–57.1) (Figure 2A). In the multivariate binary Poisson regression, the environment presented a significant influence on the concordance: The PHCC of Marovoay [APR 0.53 (95% CI 0.44–0.65)] showed a 47% lower concordance when compared to the PHCC in Ankazomborona. Additionally, the influence on the concordance was observed in the practice of the midwives; in comparison to the first 3 months of the screening, an increased practice in screening from 6 to 8 months led to a 4% higher concordance (APR 1.04); however, the confidence interval ranged from 0.93 to 1.17.

Variability in specificity was also observed in the macrodomain of procedure characteristics (Figure 2B) such as the environmental factors displayed by the PHCC, with a specificity of 16.8% (95% CI 10.2–26.2) in Marovoay in contrast to a specificity of 60.7% (95% CI 40.7–77.9) in Ankazomborona and 35.3% (95% CI 20.3–53.5) in Antanambao‐Andranolava. A further difference was observed in the specificity of the number of midwives' colposcopy training: 33.3% (95% CI 25.0–42.9) for two colposcopy trainings and 16.3% (95% CI 7.3–31.3) for one colposcopy training. While there was a small difference in specificity between a screening practice of 3–5 months [14.3% (95% CI 7.9–24.0)] and 6–8 months [18.8% (95% CI 7.9–37.0)], a screening practice of 0–2 months showed a specificity of 65.9% (95% CI 49.3–79.4). Methodological factors such as higher and lower quality pictures exhibited a specificity of 36.7% (95% CI 23.8–51.7) and 25.0% (95% CI 17.4–34.4), respectively. Among images with no FGS signs in the reference diagnosis (*n* = 157), in the environment of the PHCC in Marovoay compared to the PHCC of Ankazomborona, decreased specificity by 49% [APR 0.51 (95% CI 0.28–0.90)]. Additionally, a decrease in the specificity of 73% [APR 0.27 (95% CI 0.15–0.49)] was observed for the screening months of 3–5 months and a decrease of 63% [APR 0.37 (95% CI 0.116–0.83)] for 6–8 months, compared to the time interval of 0–2 months.

Sensitivity (Figure 2C) was 93.9% (95% CI 88.8–96.9) and 98.9% (95% CI 95.5–99.8) for the methodology indicator of higher and lower quality images, respectively [CPR 0.95 (95% CI 0.91–0.99)]. In the univariate Poisson regression, sensitivity increased slightly with study practice from 92.5% (95% CI 85.4–96.5) for 0–2 months to 98.6% (95% CI 94.4–99.8) for 3–5 months [CPR 1.07 (95% CI 1.01–1.13)].

## 4 Discussion

The present study reports an overall high sensitivity [96.4% (95% CI 93.7–98.0)] and a relatively low specificity [28.7% (95% CI 21.8–36.5)] of midwife‐led colposcopy for the detection of FGS at the primary level of care in a highly endemic country for the disease, with a fair agreement [*κ* 0.30 (95% CI 0.22–0.39)] among midwives and gynaecologists. The practice of midwives and the environment of practice were identified as factors influencing colposcopy performance. Although performance was partially recovered toward the end of the study, the negative effect of midwives' length of practice on both the ability to correctly identify negative cases and overall diagnostic concordance was observed. Additionally, the PHCC environment in Marovoay showed to negatively influence concordance and specificity.

This study shows that colposcopy in this specific setting is a very sensitive but not specific method for the detection of FGS‐related lesions, leading to a relatively high number of women (*n* = 112) detected as false positives for the condition. The most direct consequence of this feature is the unnecessary treatment offered to women following the diagnosis. FGS is generally treated with a single dose of 40 mg/kg of praziquantel. The treatment is considered safe and is recommended as a therapeutic intervention for schistosomiasis and as a preventive chemoprophylaxis in mass drug administration campaigns for children and adults, although the latter are still poorly included in the campaigns [26]. Praziquantel is mostly effective against the adult form of the parasite, hence exerting its optimal effectiveness in active schistosome infections.

In a highly endemic context such as Madagascar, the prevalence of schistosome infections among adults is extremely high [19]. In this view, even though women are incorrectly diagnosed with FGS, there remains a high likelihood that they carry a schistosome infection or are at risk of developing FGS in the future [9], making the treatment derived from an inaccurate diagnosis still a potential health benefit for the women. Additionally, the screening itself can allow the identification of further asymptomatic gynaecological disorders [27] that would remain undetected and eventually managed at advanced stages when the success of cure might be lower [28]. On the other hand, the misdiagnosis due to false positive detection of FGS might mask other infections of the vaginal tract and delay or prevent proper treatment similar to other lower genitourinary tract conditions [29]. Given the seldom contact of women in rural Madagascar with the health system, the likelihood of follow‐up visits is generally low. Nevertheless, vaginal infections are frequently symptomatic, increasing the chances to prevent missed treatments. In order to contain such consequences, colposcopy screening should be accompanied by proper counselling aimed at women to observe symptoms and to explain the results of the screening and its implications [30, 31].

This study additionally shows that the environment presents an influence on colposcopy performances. The screening environment of the PHCC of Marovoay presented a significantly decreased performance (Concordance: APR 0.53; Specificity: APR 0.51) in the detection of FGS. This PHCC is located in a peri‐urban environment with women travelling from the vicinity of the town to the PHCC for the screening. Benites‐Zapata et al. [15] report that the distance to health care facilities has an influence on the success of services provided. This might explain the decreased performances of colposcopy in Marovoay, where women normally travel longer to reach the PHCC and then could exercise (unconsciously) pressure to conclude the medical procedure quickly.

Interestingly, the proportions of false positive results increased during the course of the study, leading to a sharp decrease in specificity between the initial and following months (APR 3–5 months 0.27, APR 6–8 months 0.37) of the study. This finding aligns with similar studies that show decreasing performances of health care professionals with increasing practice [32]. It can be observed that the most evident decline occurred during the busiest timeframe of the study (3–5 months) and remained low after this time period, even though numbers of visits went down again. A high workload is one of the most common limitations of work in primary health care settings [33]. Dissemination of guidelines and training material has been previously observed to be inefficient in improving performance, while applied approaches such as coordinated mentoring showed promising improvement [34]. Supporting these concepts of the integrated mentoring approaches is that the number of trainings had no significant influence on the diagnostic performance of the midwives. However, a non‐significant trend toward the better detection of negative cases by midwives with multiple trainings was observed [APR 1.49 (95% CI 0.72–3.08)]. Midwives that did not participate in the initial training but only in the refresher training were paired with colposcopy‐experienced midwives, which allowed for targeted mentoring. Finally, no influences could be observed on the diagnostic performances from the methodological micro domain.

Though this study presents significant strengths such as the direct implementation in highly rural and real‐life settings, the highly standardised procedures and regular training, it doesn't come without limitations. This study was performed in a high endemicity area; hence, generalizability of findings should be made carefully as it might vary in different epidemiological contexts. This study did not meet the estimated sample size, though initial recruitment provided these numbers. However, the final sample size is not lower than that in similar studies [35]. Finally, this study does not provide a diagnosis of active schistosome infection in the participants. Nevertheless, the main objective of the study is to investigate the performances of colposcopy‐based FGS diagnosis at the primary level of care in comparison to an expert gynaecologist diagnosis and not to examine colposcopy as an overall diagnostic tool in comparison to other diagnostic methods.

## 5 Conclusion

In conclusion, this study demonstrates that midwives at the primary level of care in endemic settings can detect FGS with high sensitivity but limited specificity when compared to expert gynaecologists' assessment of colposcopy images. This suggests that implementing colposcopy at the primary care level for FGS screening is feasible but requires appropriate quality assurance measures. Targeted mentoring approaches and workload management may better sustain diagnostic quality.

## Ethics Statement

This study was approved by the Ethics Committee Hamburg State Medical Chamber (protocol number PV7309‐4707_2‐BO‐ff) and the National Ethics Committee of Madagascar (protocol number 052/MSANP/SG/AGMED/CERBM) and adhered to the principles embodied in the Declaration of Helsinki.

## Consent

Written informed consent was obtained from all participants included in the study. Participants had the right to withdraw informed consent and study participation at any time without giving reasons.

## Conflicts of Interest

The authors declare no conflicts of interest.

## Supporting information


**Data S1:** Supporting Information.

**Data S2:** Supporting Information.

**Table S1:** Measures of diagnostic accuracy stratified by groups.

## Data Availability

The datasets used and analysed during the current study are available from the corresponding author on reasonable request.

## References

[1] F. F. D. Teukeng , M. Blin , N. Bech , et al., “Hybridization Increases Genetic Diversity in Schistosoma Haematobium Populations Infecting Humans in Cameroon,” Infectious Diseases of Poverty 11, no. 1 (December 2022): 37. 35346375 10.1186/s40249-022-00958-0 PMC8962594

[2] D. Engels , P. J. Hotez , C. Ducker , et al., “Integration of Prevention and Control Measures for Female Genital Schistosomiasis, HIV and Cervical Cancer,” Bulletin of the World Health Organization 98, no. 9 (September 2020): 615–624. 33012861 10.2471/BLT.20.252270 PMC7463188

[3] V. N. Orish , E. K. S. Morhe , W. Azanu , R. K. Alhassan , and M. Gyapong , “The Parasitology of Female Genital Schistosomiasis,” Current Research in Parasitology & Vector‐Borne Diseases 27, no. 2 (May 2022): 100093. 10.1016/j.crpvbd.2022.100093 PMC9198370 35719849

[4] P. J. Hotez , D. Engels , M. Gyapong , C. Ducker , and M. N. Malecela , “Female Genital Schistosomiasis,” New England Journal of Medicine 381, no. 26 (2019): 2493–2495. 31881137 10.1056/NEJMp1914709

[5] WHO , “Female Genital Schistosomiasis: A Pocket Atlas for Clinical Health‐Care Professionals,” 2015, https://www.who.int/publications‐detail‐redirect/9789241509299.

[6] H. D. Mazigo , A. Samson , V. J. Lambert , et al., “Female Genital Schistosomiasis Is a Sexually Transmitted Disease,” in Gaps in Healthcare Workers’ Knowledge About Female Genital Schistosomiasis in Tanzania, vol. 2, ed. M. Kamndaya (PLOS Glob Public Health, 2022), e0000059. 10.1371/journal.pgph.0000059 PMC10021524 36962298

[7] World Health Organization and UNAIDS , “No More Neglect—Female Genital Schistosomiasis and HIV—Integrating Sexual and Reproductive Health Interventions to Improve Women's Lives,” Geneva, 2019, 44, Report No.: UNAIDS/JC2979, https://www.unaids.org/sites/default/files/media_asset/female_genital_schistosomiasis_and_hiv_en.pdf.

[8] J. M. Kutz , P. Rausche , T. Rasamoelina , et al., “Female Genital Schistosomiasis, Human Papilloma Virus Infection, and Cervical Cancer in Rural Madagascar: A Cross Sectional Study,” Infectious Diseases of Poverty 12, no. 1 (September 2023): 89. 37749705 10.1186/s40249-023-01139-3 PMC10518971

[9] O. Lamberti , F. Bozzani , K. Kiyoshi , and A. L. Bustinduy , “Time to Bring Female Genital Schistosomiasis out of Neglect,” British Medical Bulletin 149, no. 1 (March 2024): 45–59. 38220571 10.1093/bmb/ldad034 PMC10938538

[10] S. D. Holmen , E. Kleppa , K. Lillebø , et al., “The First Step Toward Diagnosing Female Genital Schistosomiasis by Computer Image Analysis,” American Journal of Tropical Medicine and Hygiene 93, no. 1 (July 2015): 80–86. 25918212 10.4269/ajtmh.15-0071 PMC4497910

[11] E. F. Kjetland , P. D. C. Leutscher , and P. D. Ndhlovu , “A Review of Female Genital Schistosomiasis,” Trends in Parasitology 28, no. 2 (February 2012): 58–65. 22245065 10.1016/j.pt.2011.10.008

[12] J. Valls , A. Baena , G. Venegas , et al., “Performance of Standardised Colposcopy to Detect Cervical Precancer and Cancer for Triage of Women Testing Positive for Human Papillomavirus: Results From the ESTAMPA Multicentric Screening Study,” Lancet Global Health 11, no. 3 (2023): e350–e360. 36796982 10.1016/S2214-109X(22)00545-9 PMC10020136

[13] K. Benkortbi , R. Catarino , A. Wisniak , et al., “Inter‐ and Intra‐Observer Agreement in the Assessment of the Cervical Transformation Zone (TZ) by Visual Inspection With Acetic Acid (VIA) and Its Implications for a Screen and Treat Approach: A Reliability Study,” BMC Women's Health 23, no. 1 (January 2023): 27. 36658551 10.1186/s12905-022-02131-z PMC9854065

[14] P. Xue , M. T. A. Ng , and Y. Qiao , “The Challenges of Colposcopy for Cervical Cancer Screening in LMICs and Solutions by Artificial Intelligence,” BMC Medicine 18, no. 1 (December 2020): 169. 32493320 10.1186/s12916-020-01613-x PMC7271416

[15] V. A. Benites‐Zapata , E. A. Hernandez‐Bustamante , L. M. Acuña‐Chávez , et al., “Colposcopy in the Primary Health Care: A Scoping Review,” Journal of Primary Care & Community Health 14 (September 2023): 21501319231198944. 10.1177/21501319231198942 PMC10517605 37740513

[16] S. Mittal , R. Mandal , D. Banerjee , et al., “HPV Detection‐Based Cervical Cancer Screening Program in Low‐Resource Setting: Lessons Learnt From a Community‐Based Demonstration Project in India,” Cancer Causes & Control 27, no. 3 (March 2016): 351–358. 26712612 10.1007/s10552-015-0708-z

[17] L. N. Pillay , I. Umbelino‐Walker , D. Schlosser , C. Kalume , and R. Karuga , “Minimum Service Package for the Integration of Female Genital Schistosomiasis Into Sexual and Reproductive Health and Rights Interventions,” Frontiers in Tropical Diseases 5 (June 2024): 1321069.

[18] M. V. Evans , T. Andréambeloson , M. Randriamihaja , et al., “Geographic Barriers to Care Persist at the Community Healthcare Level: Evidence From Rural Madagascar,” PLOS Global Public Health 2, no. 12 (2022): e0001028. 36962826 10.1371/journal.pgph.0001028 PMC10022327

[19] S. K. Gruninger , T. Rasamoelina , R. A. Rakotoarivelo , et al., “Prevalence and Risk Distribution of Schistosomiasis Among Adults in Madagascar: A Cross‐Sectional Study,” Infectious Diseases of Poverty 12, no. 1 (April 2023): 44. 37098581 10.1186/s40249-023-01094-z PMC10127445

[20] WHO Strategic and Technical Advisory Group of Experts for Maternal, Newborn, Child, and Adolescent Health and Nutrition (STAGE) , “Transforming Women's, Children's, and Adolescents’ Health and Wellbeing Through Primary Health Care,” Lancet 402, no. 10413 (2023): 1606–1608. 37722398 10.1016/S0140-6736(23)01909-8

[21] P. A. Harris , R. Taylor , B. L. Minor , et al., “The REDCap Consortium: Building an International Community of Software Platform Partners,” Journal of Biomedical Informatics 95 (July 2019): 103208. 31078660 10.1016/j.jbi.2019.103208 PMC7254481

[22] P. A. Harris , R. Taylor , R. Thielke , J. Payne , N. Gonzalez , and J. G. Conde , “Research Electronic Data Capture (REDCap)—A Metadata‐Driven Methodology and Workflow Process for Providing Translational Research Informatics Support,” Journal of Biomedical Informatics 42, no. 2 (April 2009): 377–381. 18929686 10.1016/j.jbi.2008.08.010 PMC2700030

[23] S. Perez‐Jaume , K. Skaltsa , N. Pallarès , and J. L. Carrasco , “ThresholdROC : Optimum Threshold Estimation Tools for Continuous Diagnostic Tests in R,” Journal of Statistical Software 82, no. 4 (2017): 1–21, http://www.jstatsoft.org/v82/i04/.

[24] J. R. Landis and G. G. Koch , “The Measurement of Observer Agreement for Categorical Data,” Biometrics 33, no. 1 (1977): 159–174. 843571

[25] D. Meyer , A. Zeileis , K. Hornik , F. Gerber , and M. Friendly , “Package ‘vcd’,” 2025, https://cran.r‐project.org/web/packages/vcd/vcd.pdf.

[26] WHO , WHO Guideline on Control and Elimination of Human Schistosomiasis (World Health Organization, 2022), https://apps.who.int/iris/handle/10665/351856. 35235279

[27] V. Marchese , Z. Rakotomalala , J. M. Kutz , et al., “Case Report: Three Cases of Suspected Female Genital Schistosomiasis and Precancerous Lesions for Cervical Cancer in a Highly Endemic Country—From Clinical Management to Public Health Implications,” Frontiers in Tropical Diseases 4 (October 2023): 1270852.

[28] A. Shiraz , R. Crawford , N. Egawa , H. Griffin , and J. Doorbar , “The Early Detection of Cervical Cancer. The Current and Changing Landscape of Cervical Disease Detection,” Cytopathology 31, no. 4 (2020): 258–270. 32301535 10.1111/cyt.12835

[29] L. Kuritzky , Z. Huynh , R. Arcenas , et al., “Potential Delayed and/or Missed STI Diagnoses Among Outpatients Presenting With Lower Genitourinary Tract Symptoms: A Real‐World Database Study,” Postgraduate Medicine 135, no. 8 (November 2023): 809–817. 37961909 10.1080/00325481.2023.2280439

[30] N. R. Bell , G. Thériault , H. Singh , and R. Grad , “Measuring What Really Matters: Screening in Primary Care,” Canadian Family Physician 65, no. 11 (2019): 790–795. 31722909 PMC6853363

[31] M. Almonte , R. Murillo , G. I. Sánchez , et al., “Multicentric Study of Cervical Cancer Screening With Human Papillomavirus Testing and Assessment of Triage Methods in Latin America: the ESTAMPA Screening Study Protocol,” BMJ Open 10, no. 5 (May 2020): e035796. 10.1136/bmjopen-2019-035796 PMC7252979 32448795

[32] R. Sankaranarayanan , B. M. Nene , K. A. Dinshaw , et al., “A Cluster Randomized Controlled Trial of Visual, Cytology and Human Papillomavirus Screening for Cancer of the Cervix in Rural India,” International Journal of Cancer 116, no. 4 (September 2005): 617–623. 15818610 10.1002/ijc.21050

[33] T. V. Nesengani , C. Downing , and W. ten Ham‐Baloyi , “Barriers to Effective Patient Care as Experienced by Nurses in Primary Healthcare Clinics in African Countries: A Systematic Review of Qualitative Studies,” BMC Nursing 24, no. 1 (February 2025): 232. 40022067 10.1186/s12912-025-02877-5 PMC11869662

[34] A. Vasan , D. C. Mabey , S. Chaudhri , H. A. Brown Epstein , and S. D. Lawn , “Support and Performance Improvement for Primary Health Care Workers in Low‐ and Middle‐Income Countries: A Scoping Review of Intervention Design and Methods,” Health Policy and Planning 32 (December 2016): czw144–czw452. 10.1093/heapol/czw144 PMC5400115 27993961

[35] A. Sturt , H. Bristowe , E. Webb , et al., “Visual Diagnosis of Female Genital Schistosomiasis in Zambian Women From Hand‐Held Colposcopy: Agreement of Expert Image Review and Association With Clinical Symptoms,” Wellcome Open Research 8 (2023): 14. 36864924 10.12688/wellcomeopenres.18737.1 PMC9971661

