# Supplementary material for: Evaluation of Midwife‐Led Colposcopy for Female Genital Schistosomiasis Screening at Primary Level of Care in Rural Madagascar: A Cross‐Sectional Study
Source: Trop Med Int Health. 2025 Oct 30;31(1):49–57. doi: 10.1111/tmi.70049 (PMC12775888; doi:10.1111/tmi.70049)
Supplement: Supplementary file 1 — Data S1: Supporting Information. [file TMI-31-49-s001.docx]

**A. Vérifier les critères**

| A01 | Répond aux critères d’inclusion ? ◯ non ◯ oui | Si non 🡪EXCLUSION |
| --- | --- | --- |
| A02 | Le consentement éclairé a été donné pour participer à l’étude ?  ◯ non ◯ oui | Si non 🡪EXCLUSION |
| A03 | Le contentement pour l’utilisation des échantillons pour  d’autres études a été signé ? ◯ non ◯ oui | Pas d’effet (continuer) |

**B. Identification et données personnelles**

| B01 | Avez-vous déjà passé un entretien FIRM-UP ? ◯ non ◯ oui ◯ oui, mais PID inconnu  🡪 Si oui, PID : FGS-\|__\|__\|__\|__\|__\|-BL |
| --- | --- |
| B02 | Nom du CSB/lieu de recrutement :  ◯ Antanambao Andranolava ◯ Ankazomborona ◯ Marovoay |
| B03 | Date de l’entretien : \| _j_ \| _j_ \|/\| _m_ \| _m_ \|/\| _a_ \| _a_ \| |
| B04 | Âge : \|__\|__\| ans |

**C. Histoire clinique**

| C01 | Avez-vous déjà été traitée avec du Praziquantel (traitement de Schisto) ?  ◯ non ◯ oui ◯ inconnu  🡪 Si oui : 🞏 la semaine passée 🞏 il y a 3 mois 🞏 il y a 6 mois  🞏 il y a un an 🞏 il y a plus d’un an 🞏 inconnu | | | | | | |
| --- | --- | --- | --- | --- | --- | --- | --- |
| C05 | Avez-vous remarqué … | non | dans le dernier mois | dans les derniers  3 mois | dans les derniers  6 mois | dans plus de  6 mois | inconnu |
|  | saignement irrégulier ? | 🞏 | 🞏 | 🞏 | 🞏 | 🞏 | 🞏 |
|  | des pertes vaginales ? | 🞏 | 🞏 | 🞏 | 🞏 | 🞏 | 🞏 |
|  | un prurit génital ou une sensation de brûlure? | 🞏 | 🞏 | 🞏 | 🞏 | 🞏 | 🞏 |
|  | une douleur pendant/après les rapports sexuels? | 🞏 | 🞏 | 🞏 | 🞏 | 🞏 | 🞏 |

**E. Diagnostics**

| E03 | Colposcopie faite ? ◯ non ◯ oui  🡪 Si oui, résultat : 🞏 Normal  🞏 Signes d'inflammation ou d'infection  🞏 Signes de la bilharziose féminine génitale (FGS)  🞏 Signes de changements précancéreux ou cancéreux  🞏 Myome  🞏 Polype  🞏 inconnu  🞏 autre : __________________________________________________  __________________________________________________________  🡪 Si non, pourquoi : ◯ rupture de stock ◯ rupture d'électricité ◯ la femme refuse  ◯ autre : ___________________________________________________ |
| --- | --- |

**Y. Remarques**

| __________________________________________________________________________________  __________________________________________________________________________________  __________________________________________________________________________________ |
| --- |

**Z. Responsabilités**

|  | Rôle dans l’étude | Initiales | Signature | Date de signature |
| --- | --- | --- | --- | --- |
| Z01 | Infirmière d’étude | \|__\|__\|__\| | _________________ | \| _j_ \| _j_ \|/\| _m_ \| _m_ \|/\| _a_ \| _a_ \| |
| Z02 | Saisie des données : 1ère | \|__\|__\|__\| | _________________ | \| _j_ \| _j_ \|/\| _m_ \| _m_ \|/\| _a_ \| _a_ \| |
| Z03 | Saisie des données : 2ème | \|__\|__\|__\| | _________________ | \| _j_ \| _j_ \|/\| _m_ \| _m_ \|/\| _a_ \| _a_ \| |
| Z04 | Responsable qualité | \|__\|__\|__\| | _________________ | \| _j_ \| _j_ \|/\| _m_ \| _m_ \|/\| _a_ \| _a_ \| |
