# Supplementary material for: Evaluation of Midwife‐Led Colposcopy for Female Genital Schistosomiasis Screening at Primary Level of Care in Rural Madagascar: A Cross‐Sectional Study
Source: Trop Med Int Health. 2025 Oct 30;31(1):49–57. doi: 10.1111/tmi.70049 (PMC12775888; doi:10.1111/tmi.70049)
Supplement: Supplementary file 2 — Data S2: Supporting Information. [file TMI-31-49-s003.docx]

Supplementary 2: STARD Checklist

# Evaluation of midwife-led colposcopy for Female Genital Schistosomiasis screening at primary level of care in rural Madagascar: A cross-sectional study

Pia Rausche(ORCiD: 0009-0002-3554-617X)^1,2^, Jean-Marc Kutz ^1,2^, Zoly Rakotomalala^3^, Bodo Sahondra Randrianasolo^4^ (ORCiD 0000-0003-4408-1482), Paule Donven^1,2^ (ORCiD 0000-0003-2740-6505), Rivo Solotiana Rakotomalala^3^, Alexina Olivasoa Tsiky Zafinimampera^5^, Olivette Totofotsy^5^, Sonya Ratefiarisoa^5^, Ravo Razafindrakoto^6^ (ORCHiD 0009-0004-1355-5889), Nantenaina Matthieu Razafindralava^6^, Zaraniaina Tahiry Rasolojaona^6^(ORCiD 0000-0002-9056-4138), Jana Hey^1,2^ (ORCiD 0009-0008-1848-0096), Aaron Remkes^1,2^ (ORCiD 0000-0003-0229-1991), Tahinamandranto Rasamoelina^6^ (ORCiD 0000-0001-7814-8767), Eva Lorenz^2,7^ (ORCiD 0000-0002-6057-0078), Rapahel Rakotozandrindrainy^8^ (ORCiD 0000-0001-8100-8626), Jürgen May^2,7,9^ (ORCiD 0000-0001-7831-8420), Monika Hampl^10,11^ (ORCiD 0000-0002-5949-5891), Irina Kislaya^2,7^ (ORCiD 0000-0001-5772-2416), Valentina Marchese^1,2^ (ORCHiD 0000-0002-5221-1614), Rivo Andry Rakotoarivelo^5^ (ORCiD 0000-0003-3438-8624), Daniela Fusco^1,2^ (ORCiD 0000-0001-8833-239X)

^1^ RG Implementation Research, Bernhard Nocht Institute for Tropical Medicine, Hamburg, Germany

^2^ German Center for Infection Research (DZIF), Hamburg-Borstel-Lübeck-Riems, Germany

^3^ Centre Hospitalier Universitaire Androva, Mahajanga, Madagascar

^4^ Association K’OLO VANONA, Antananarivo, Madagascar

^5^ University Fianarantsoa, Fianarantsoa, Madagascar

^6^ Centre d’Infectiologie Charles Mérieux, Antananarivo, Madagascar

^7^ Department of Infectious Diseases Epidemiology, Bernhard Nocht Institute for Tropical Medicine, Hamburg, Germany

^8^ University Antananarivo, Antananarivo, Madagascar

^9^ Tropical Medicine I, University Medical Center Hamburg-Eppendorf (UKE), Hamburg, Germany

^10^ Department of Obstetrics and Gynaecology, St. Elisabeth Hospital, Cologne-Hohenlind, Germany

^11^ University Hospital of Düsseldorf, Düsseldorf, Germany

*** Correspondence:**Daniela Fusco
fusco@bnitm.de

Supplementary 2: STARD Checklist

|  | **Section & Topic** | **No** | **Item** | **Reported on page #** |
| --- | --- | --- | --- | --- |
|  |  |  |  |  |
|  | **TITLE OR ABSTRACT** |  |  |  |
|  |  | **1** | Identification as a study of diagnostic accuracy using at least one measure of accuracy  (such as sensitivity, specificity, predictive values, or AUC) | 3 |
|  | **ABSTRACT** |  |  |  |
|  |  | **2** | Structured summary of study design, methods, results, and conclusions  (for specific guidance, see STARD for Abstracts) | 3 |
|  | **INTRODUCTION** |  |  |  |
|  |  | **3** | Scientific and clinical background, including the intended use and clinical role of the index test | 5 |
|  |  | **4** | Study objectives and hypotheses | 6 |
|  | **METHODS** |  |  |  |
|  | *Study design* | **5** | Whether data collection was planned before the index test and reference standard  were performed (prospective study) or after (retrospective study) | 6-8 |
|  | *Participants* | **6** | Eligibility criteria | 6 |
|  |  | **7** | On what basis potentially eligible participants were identified  (such as symptoms, results from previous tests, inclusion in registry) | 6 |
|  |  | **8** | Where and when potentially eligible participants were identified (setting, location and dates) | 6 |
|  |  | **9** | Whether participants formed a consecutive, random or convenience series | 6 |
|  | *Test methods* | **10a** | Index test, in sufficient detail to allow replication | 7 |
|  |  | **10b** | Reference standard, in sufficient detail to allow replication | 7-8 |
|  |  | **11** | Rationale for choosing the reference standard (if alternatives exist) | 5-6, 8 |
|  |  | **12a** | Definition of and rationale for test positivity cut-offs or result categories  of the index test, distinguishing pre-specified from exploratory | 7 |
|  |  | **12b** | Definition of and rationale for test positivity cut-offs or result categories  of the reference standard, distinguishing pre-specified from exploratory | 8 |
|  |  | **13a** | Whether clinical information and reference standard results were available  to the performers/readers of the index test | 7 |
|  |  | **13b** | Whether clinical information and index test results were available  to the assessors of the reference standard | 8 |
|  | *Analysis* | **14** | Methods for estimating or comparing measures of diagnostic accuracy | 8 |
|  |  | **15** | How indeterminate index test or reference standard results were handled | 8 |
|  |  | **16** | How missing data on the index test and reference standard were handled | 8 |
|  |  | **17** | Any analyses of variability in diagnostic accuracy, distinguishing pre-specified from exploratory | 9 |
|  |  | **18** | Intended sample size and how it was determined | 9 |
|  | **RESULTS** |  |  |  |
|  | *Participants* | **19** | Flow of participants, using a diagram | 9, Figure 1 |
|  |  | **20** | Baseline demographic and clinical characteristics of participants | 9 + Table 1 |
|  |  | **21a** | Distribution of severity of disease in those with the target condition | NA |
|  |  | **21b** | Distribution of alternative diagnoses in those without the target condition | NA |
|  |  | **22** | Time interval and any clinical interventions between index test and reference standard | NA |
|  | *Test results* | **23** | Cross tabulation of the index test results (or their distribution)  by the results of the reference standard | Table 2 |
|  |  | **24** | Estimates of diagnostic accuracy and their precision (such as 95% confidence intervals) | 10 |
|  |  | **25** | Any adverse events from performing the index test or the reference standard | NA |
|  | **DISCUSSION** |  |  |  |
|  |  | **26** | Study limitations, including sources of potential bias, statistical uncertainty, and generalisability | 12-13 |
|  |  | **27** | Implications for practice, including the intended use and clinical role of the index test | 11-13 |
|  | **OTHER INFORMATION** |  |  |  |
|  |  | **28** | Registration number and name of registry | 2 |
|  |  | **29** | Where the full study protocol can be accessed | NA |
|  |  | **30** | Sources of funding and other support; role of funders | 2 |
|  |  |  |  |  |
