# Supplementary material for: Evaluation of Midwife‐Led Colposcopy for Female Genital Schistosomiasis Screening at Primary Level of Care in Rural Madagascar: A Cross‐Sectional Study
Source: Trop Med Int Health. 2025 Oct 30;31(1):49–57. doi: 10.1111/tmi.70049 (PMC12775888; doi:10.1111/tmi.70049)
Supplement: Supplementary file 3 — Table S1: Measures of diagnostic accuracy stratified by groups. [file TMI-31-49-s002.docx]

Supporting Table 1: Measures of diagnostic accuracy stratified by groups

|  |  | *Reference* | | **Concordance**  **(95% CI)** | **Sensitivity**  **(95% CI)** | **Specificity**  **(95% CI)** | **PPV**  **(95% CI)** | **NPV**  **(95% CI)** |
| --- | --- | --- | --- | --- | --- | --- | --- | --- |
|  | *Midwife* | **+** | **-** |  |  |  |  |  |
| **Participant characteristics** |  |  |  |  |  |  |  |  |
| **Age group** |  |  |  |  |  |  |  |  |
| 18-27 years | **+** | 157 | 58 | 74.1 | 96.3 | 31.0 | 73.0 | 81.2 |
|  | **-** | 6 | 26 | (68.2- 79.4) | (91.8- 98.5) | (21.6- 42.1) | (66.5- 78.7) | (63.0- 92.1) |
| 28-37 years | **+** | 88 | 31 | 73.2 | 94.6 | 24.4 | 73.9 | 66.7 |
|  | **-** | 5 | 10 | (64.8- 80.4) | (87.3- 98.0) | (12.9- 40.6) | (65.0- 81.4) | (38.7- 87.0) |
| 38+ years | **+** | 81 | 23 | 79.0 | 98.8 | 28.1 | 77.9 | 90.0 |
|  | **-** | 1 | 9 | (70.3- 86.0) | (92.5- 99.9) | (14.4- 47.0) | (68.5- 85.2) | (54.1- 99.5) |
| **Gynaecological Symptoms** |  |  |  |  |  |  |  |  |
| No symptoms | **+** | 106 | 38 | 73.5 | 95.5 | 25.5 | 73.6 | 72.2 |
|  | **-** | 5 | 13 | (66.0- 80.1) | (89.3- 98.3) | (14.8- 39.9) | (65.5- 80.4) | (46.4- 89.3) |
| Symptoms | **+** | 220 | 74 | 75.7 | 96.9 | 30.2 | 74.8 | 82.1 |
|  | **-** | 7 | 32 | (70.6-80.1) | (93.4-98.6) | (21.9- 40.0) | (69.4- 79.6) | (65.9- 91.9) |
| **Procedure characteristics** |  |  |  |  |  |  |  |  |
| **Enviromental** |  |  |  |  |  |  |  |  |
| Marovoay | **+** | 61 | 79 | 49.0 | 98.4 | 16.8 | 43.6 | 94.1 |
|  | **-** | 1 | 16 | (41.0- 57.1) | (90.2- 99.9) | (10.2- 26.2) | (35.3- 52.2) | (69.2- 99.7) |
| Ankazomborona | **+** | 128 | 11 | 88.4 | 94.1 | 60.7 | 92.1 | 68.0 |
|  | **-** | 8 | 17 | (82.5- 92.9) | (88.4-97.2) | (40.7- 77.9) | (86.0- 95.8) | (46.5-84.3) |
| Antanambao-Andranolava | **+** | 137 | 22 | 85.6 | 97.9 | 35.3 | 86.2 | 80.0 |
|  | **-** | 3 | 12 | (79.5- 90.5) | (93.4- 99.4) | (20.3- 53.5) | (79.6- 90.9) | (51.4-90.9) |
| **Training** |  |  |  |  |  |  |  |  |
| 2 trainings | **+** | 269 | 76 | 78.1 | 96.4 | 33.3 | 78.0 | 79.2 |
|  | **-** | 10 | 38 | (73.7- 82.1) | (93.3-98.2) | (25.0-42.9) | (73.1- 82.2) | (64.6- 89.0) |
| 1 training | **+** | 57 | 36 | 62.8 | 96.6 | 16.3 | 61.3 | 77.8 |
|  | **-** | 2 | 10 | (53.9- 73.0) | (87.3-99.4) | (7.3-31.3) | (50.6- 71.0) | (40.2- 96.1) |
| **Practice** |  |  |  |  |  |  |  |  |
| 0,1,2 | **+** | 99 | 14 | 85.1 | 92.5 | 65.9 | 87.6 | 77.1 |
|  | **-** | 8 | 27 | (78.4- 90.4) | (85.4-96.5) | (49.3-79.4) | (79.8- 92.8) | (59.4- 89.0) |
| 3,4,5 | **+** | 138 | 72 | 67.0 | 98.6 | 14.3 | 65.7 | 85.7 |
|  | **-** | 2 | 12 | (60.4- 73.1) | (94.4-99.8) | (7.9-24.0) | (58.8- 72.0) | (56.2- 97.5) |
| 6,7,8 | **+** | 89 | 26 | 77.3 | 97.8 | 18.8 | 77.4 | 75.0 |
|  | **-** | 2 | 6 | (68.8- 84.3) | (91.5-99.6) | (7.9-37.0) | (68.5-84.4) | (35.6- 95.5) |
| **Methodological** |  |  |  |  |  |  |  |  |
| Lower quality | **+** | 172 | 81 | 70.6 | 98.9 | 25.0 | 68.0 | 93.1 |
|  | **-** | 2 | 27 | (64.8-75.7) | (95.5-99.8) | (17.4-34.4) | (61.8-73.6) | (75.8-98.8) |
| Higher quality | **+** | 154 | 31 | 80.8 | 93.9 | 36.7 | 83.2 | 64.3 |
|  | **-** | 10 | 18 | (74.7-85.7) | (88.8-96.9) | (23.8-51.7) | (76.9-88.2) | (44.1-80.7) |
